# Current status of *Schistosoma mansoni* infection among previously treated rural communities in the Abbey and Didessa Valleys, Western Ethiopia: Implications for sustainable control

**DOI:** 10.1371/journal.pone.0247312

**Published:** 2021-02-25

**Authors:** Alemayehu Assefa, Berhanu Erko, Svein Gunnar Gundersen, Girmay Medhin, Nega Berhe

**Affiliations:** 1 University of Assosa, College of Health Science, Assosa, Ethiopia; 2 Akililu Lemma Institute of Pathobiology, Addis Ababa University, Addis Ababa, Ethiopia; 3 Department of Global Development and Planning, University of Agder, Agder, Norway; Federal University of Agriculture, Abeokuta, NIGERIA

## Abstract

**Background:**

*Schistosoma* constitutes a major public health problem and developmental challenges in the majority of developing and subtropical regions. The World Health Organization has set guidelines for the control and elimination of schistosomiasis. Ethiopia is providing school-based Mass Drug Administration (MDA) at the study areas of the Abbey and Didessa Valleys of western Ethiopian since 2015. Moreover, mass treatment was already done in the same villages 30 years ago. However, the current *Schistosoma mansoni* infection status among humans and snails in the study areas is not known. Hence, the present study aims to determine the current status.

**Methods:**

A community-based cross-sectional study was conducted in the three communities; Chessega, Agallu Metti and Shimala in *Schistosoma mansoni* endemic areas of the Abbey and Didessa valleys in Western Ethiopia. Using the list of households obtained from the Kebele administration, a systematic sampling technique was used to select households in each village.

**Results:**

Even though the area is under the Ethiopian national Mass Drug Administration campaign, the present study reports prevalence above 50%. Although the majority of the infections were moderate, we found that 13% had heavy infection, above 400 eggs per gram of stool, which is at the same level as before the treatment campaign 30 years ago. The infection was significantly higher among those below 12 years of age, among non-attending school-age children and daily laborers.

**Conclusion:**

*Schistosoma mansoni* infection is still a public health problem in the study areas, despite control efforts already 30 years ago and present mass treatment in the last years. We suggest making the mass treatment campaign just early after the rainy season, when the snails are washed away. This should be supplemented with provisions of clean water, sanitation, and hygiene (WASH) and reduction of water contact and possible snail control efforts’ to prevent reinfection.

## Introduction

Intestinal parasitic infections, particularly schistosomiasis, pose considerable public health and development challenges in most developing tropical and subtropical regions of the world [1].

Schistosomiasis, a major parasitic disease was estimated to affect more than 240 million people worldwide, with 700 million people being at risk of infection and 20 million of whom suffer serious health consequences. About 90% of these occur in sub-Saharan Africa (SSA) [2], where infection with *S*. *mansoni* and *S*. *haematobium* are predominant [3].

The World Health Organization (WHO) has recommended guidelines for the control or elimination of schistosomiasis [4]. Schistosomiasis control has focused on treating populations via Mass Drug Administration (MDA) with praziquantel as a community- or school-based treatment program. World Health Organization (WHO) recommends using the prevalence of infection in school-age children (SAC) to determine the treatment frequency in a given endemic area [5]. The recommended treatment strategy for *Schistosoma* infection is depending upon whether the community has low (<10%), moderate (10 to <50%), or a high (50% and above) SAC prevalence at baseline. The strategy for low prevalence communities is to treat all SAC once every 3 years and treat suspected cases; moderate prevalence communities to treat all SAC and at-risk adults once every 2 years, and for high prevalence communities to treat all SAC and at-risk adults annually. The WHO guidelines suggest that after 5–6 years of MDA, with a continuously achieved coverage level of >75%, the treatment frequency may be reduced accordingly. If the prevalence remains low for 4 years following a lower treatment frequency, the treatment frequency may be further reduced. Conversely, if the prevalence returns to baseline levels, the previous treatment frequency should be reintroduced [4].

By 2020, WHO was planned to treat 75% of SAC at risk in endemic countries [6]. However, young adults (25–30 years age) also make up a large proportion of those infected. Hence, if MDA is only targeted at SAC, a large fraction of the local *Schistosoma* infection burden remains a source of infection [7].

The WHO 2020 target was “morbidity control” by reducing the prevalence of heavy-intensity infections to ≤5% among SAC. After this goal is reached, the next target for 2025 is “elimination as a public health problem,” meaning that the treated region has reached ≤1% prevalence of heavy-intensity infections among SAC by 2025 [5]. There has been a concerted international action to control and even eradicate schistosomiasis by the mass drug administration of praziquantel, the drug presently having an effect on all *Schistosoma* strains [8]. Recently, however, the SCORE (Schistosomiasis Consortium for Operational Research and Evaluation) consortium containing the forefront of schistosomiasis researchers and research institution in the world has published a summary indicating that MDA alone might not be the final solution for the eradication of schistosomiasis [9].

In Ethiopia, schistosomiasis is one of the prevalent parasitic diseases reported across many regions, causing considerable morbidity, with over five million people estimated to be infected, and more than 37 million people at the risk of infection [1]. Ethiopia has a national schistosomiasis MDA program [10]. The intestinal schistosomiasis caused by *S*. *mansoni* is endemic in most parts of the country, including the Abbey and Didessa River Basin [10, 11].

Schistosomiasis is a waterborne zoonotic parasitic disease, and the construction of large-scale water conservancy projects in schistosomiasis endemic areas will undoubtedly cause environmental changes, which may impact the diffusion of intermediate hosts and the spread of schistosomiasis [12]. The Grand Ethiopian Renaissance Dam (GERD) is under construction in the western part of the Ethiopian Abbey River. There are a number of known *Schistosome* infected rivers in this area which are tributaries of the lake that will be formed by the dam [13]. As a consequence, there will be a series of changes in the ecological environment of the marshland along the rivers and the adjacent areas of the lake. This will probably affect schistosomiasis prevalence. The lake will increase the water-related activities of people in the area and correspondingly increase the risk of schistosomiasis infection [12].

**Fig 1:**
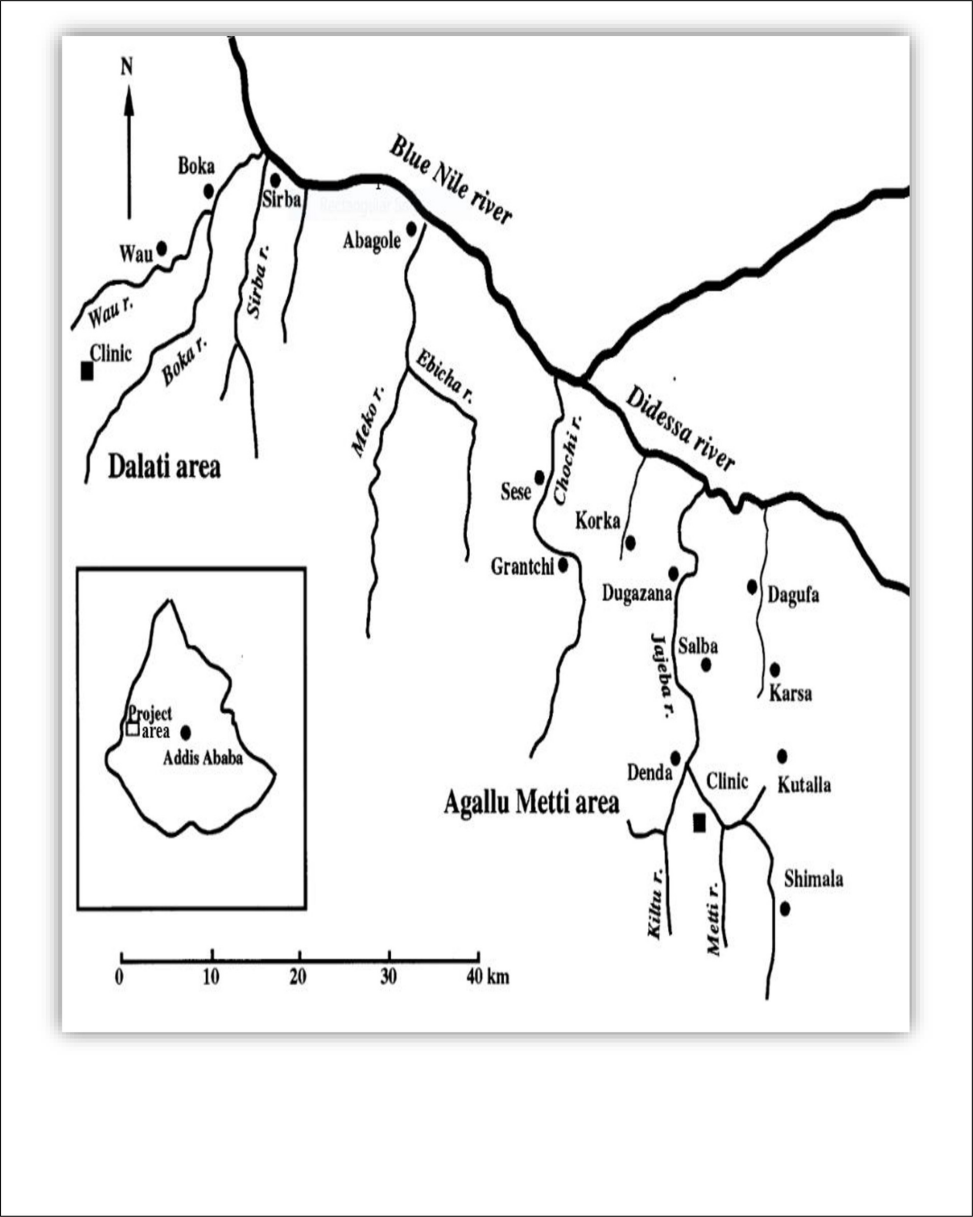
Map of the study areas.

In 1982 the overall prevalence of *S*. *mansoni* infection in this area of the Abbey valley was 27.9% (60/215) [11]. An intervention by Gundersen et al from 1985–1987, consisting of drug administration and selected focal snail control during 2 years in the endemic area brought the infection rates down from 42.4% to 11.4% in the Dalati-Sirba area and from 65.7% to 7.8% in the Agallu Metti area [13]. For map see Fig 1. In the first years after the intervention, the infection rates of humans were kept low (less than 10%) and 45% had less than 100 eggs per gram of faces. The snail infections were also dramatically reduced. There was no intervention from 1987 to 2014 in these endemic areas. Ten years after the intervention the infection of humans had risen to almost the pre-control prevalence, but still, there were very few who had an intensity of infection above 100 eggs per gram [14]. Based on information from the local health office and national NTDs control program, later schistosomiasis MDA by the local government started in 2015 and since then have been provided annually in schools, for those 5–19 years of age [10, 15]. Hence, the present study aims to determine the current status of *S*.*mansoni* infection among human and snail hosts in the Abbey and Didessa Valleys, western Ethiopia.

## Methodology

### Study area and population

For maps see Fig 1, first published in 1990 by Gundersen et al [14]. The study was conducted in three villages: The west most Chessega village, established at the same place as Wau River of the Dalati area of the Abbey Valley of the map. Chessega presently contains the population of previously examined villages of Sirba and Boka, who moved there because of the rising water level of the GERD Lake. The other villages of Metti and Shimala in the adjacent Agallu Metti area are situated eastwards on the hilly slopes south of the junction between the Didessa and Abbey rivers (700–1200 m altitude). The area belongs to the Benishangul Gumuz regional state, one of the nine regions in Ethiopia. Both areas are the extensions of Sudanese savannah with a hot, dry climate and seasonal rains from last May to early October.

The area is about 700 km west of Addis Ababa. It is mostly inhabited by a Nilotic ethnic group known as Gumuz. The total population of the three study villages according to the last census was about 8375, of which 2660 lived in Sirba Abbey (Chessega), 3800 lived in Metti and 1915 lived in Shimala (Agallu Metti). All of the inhabitants were living under similar poor environmental sanitation and low socio-economic conditions. The inhabitants of the study area earn their living as small scale farming by traditional farming method, most commonly manually using a hoe. Maize and millet are the most common crops cultivated by the community.

### Study design

A community based cross-sectional study was conducted among the three communities that live in *Schistosoma mansoni* endemic areas in the Abbey and Didessa valleys in western Ethiopia.

### Sample size determination and sampling of study participants

In a previous school-based cross-sectional study in the same area, the prevalence of *S*. *mansoni* infection was 63 to 68% [14]. Using this as the best available estimate for the prevalence of *S*.*mansoni* (63%) in the target population of the study area with 95% confidence in the estimate and assuming a 5% margin of error and 5% non-response, the minimum number of participants required for a representative study was 376.

A systematic sampling technique was employed in each of the three villages to recruit the households. For each household, we recruited one individual who full-filled the following inclusion criteria: (I) age 5 to 35 years; (II) an adult, parents or guardians, who could give written informed consent; (III) children who gave assent; (IV) individuals who lived in the study area for more than a year, and not having any critical medical illness at the time of data collection was included. Hence, Health Extension Workers (HEWs) visited the selected households, administered the consenting process and filled the questioners that document the background characteristics and invited them to come to sample collection centers and gave samples.

### Exclusion criteria

Community members who had a history of anti-helminthic medication within two months prior to registration; children younger than 5 years and adults older than 35 years; individuals who lived in the areas for less than a year, and individuals who were not willing to provide consent was excluded from the study.

### Stool sample collection and parasitological examination

The study participants were given a clean leak proof plastic sheet and clean wooden applicator and informed to bring sizable fresh, uncontaminated stool sample of their own. Stool containers were then collected and labeled with identification numbers and three Kato-Katz thick smears were prepared from each specimen as previously described [16]. The prepared Kato-Katz cellophane thick smears were examined within an hour not to miss the ova of hookworm and on the same and subsequent days for *S*.*mansoni* and other Soil Transmitted Helminths (STHs).

Quantification of eggs was done by counting the number of eggs on a smear of about 41.7mg of stool using the Keto-Katz method [16]. The mean number of eggs from each Kato-Katz thick smear was multiplied by a factor of 24 in order to express infection intensities as the number of eggs per gram of feces (Epg). The intensity of infections was described as light (1–99), moderate (100–399) and heavy (>400) eggs per gram of stool [16].

### Malacological study

A survey for *Biomphalaria pfeifferi* snail vectors was done, from common water contact sites where people collect water, wash clothes, bathe, and swim. Snail sampling was undertaken in mid-January, mid-March, and mid-May by two experienced field collectors using scoops and forceps for the duration of 30 minutes per main human contact site of each water body and was performed between 8:30 AM and 10:30 AM. The water bodies were the same as investigated in the 1995–97 [13]: Metti River, Jajeba River, and Sumala Stream at Agallu Metti and Wau River at Chessega (Sirba Abbey). During snail collection, physical characteristics of the habitat were observed, such as vegetation abundance, and water turbidity. The collected snail specimens were transferred into perforated plastic boxes and appropriately labeled and transported to the Aklilu Lemma Institute of Pathobiology (ALIPB) laboratory, Addis Ababa, where they were processed. *Biomphalaria* pfeifferi snail vectors were identified based on their shell morphology and structure using standard identification keys [17]. At 11:00 AM, snails were rinsed and placed individually on 24-well culture plates containing 1ml of clear, filtered de-chlorinated water and were exposed to indirect sunlight for 4 hours to induce cercarial shedding. The time of cercariae shedding was carefully selected to coincide with the early peak shedding time (midday) [18]. The wells of the plates were then examined for the presence of cercariae under a dissecting microscope [19].

### Quality assurance

The amount of the stool and the absence of contamination with soil was closely supervised during the sample collection. Standard Operating Procedures (SOPs) were strictly followed during the course of sample collection, transportation, processing, and examination of Kato-Katz thick smears. The findings of each laboratory test were recorded carefully. All the Kato-Katz thick smears were re-examined by the experienced laboratory technologist and the discordant slides were also re-checked by the third person at the Aklilu Lemma Institute of Pathobiology (ALIPB) Addis Ababa University. Finally, Kato-Katz thick smears read positively by two laboratory technologist were reported as positive.

### Data analysis

Data was entered in EpiData version 3.3 software and imported into IBM SPSS version 25 statistical software for statistical analyses. Descriptive statistic was used to summarize the data. Chi-square test was carried out to see the relationships or associations of different variables. The P-value of below 5% was used as a statistically significant point for all.

### Ethics approval and consent to participate

The study was ethically approved by the Institutional Review Board of Aklilu Lemma Institute of Pathobiology, Addis Ababa University (Ref No: ALIPB/IRB/002/2017/18) and a support letter was obtained from District Health Offices. Prior to conducting the study, meetings were held with the parents or guardians and community leaders to explain the aims of the study and procedures of sample collection. Moreover, the aim of the study was explained to the study participants, and written and verbal consent was obtained from the participants and /or their parents and assent for children younger than 12 years age. Those individuals who were found positive or found to have medical problems were treated and /or referred to the health facilities according to national guidelines.

## Results

### Socio-demographic characteristics

From the total planed sample size (376), results are reported for 373, making the non-response of two and one incomplete data. An almost equal number of males and females with a mean age of 10.7 years (range: 6 to 35 years) have participated in the study. The mean weight was 45.21kg and the mean height was 156.77cm. About 85% of the participants’ were Gumuz ethnic group and the educational status of the majority (64.3%), was primary. Most of the study participants were students and farmers. About 4% of study participants were non-attending school-age children (Table 1).

**Table 1:** Socio-demographic characteristics of study participants (n = 373).

Socio-demographic characteristics	N (%)
Sex	
Male	193 (51.7)
Female	180 (48.3)
Age categories	
5–12	77 (20.6)
13–19	139 (37.3)
20–35	157 (42.1)
Ethnic group	
Gumuz	317 (85.0)
Oromo	44 (11.8)
Amhara	10 (2.7)
Berta	1 (0.3)
Tigre	1 (0.3)
Educational status of participants	
Non-attending school age children	16 (4.3)
Primary	240 (64.3)
Secondary	70 (18.8)
Collage and above	32 (8.6)
Not formally educated	15 (4.0)
Participant occupation	
Farmer	73 (19.6)
Student	240 (64.3)
Salaried employee	40 (10.7)
Non-attending school age children	16 (4.3)
Day laborer	4 (1.1)
Family/household occupation	
Farmer	318 (85.3)
Merchant	6 (1.6)
Salaried employee	44 (11.8)
Day laborer	5 (1.3)

### Deworming coverage during the last mass treatment campaign among targeted age group (5–19 years age)

According to present study, the deworming coverage of the last mass treatment campaign among targeted (5–19 years age) study participants was 62.5%, of which, 65% among 5–12 years age and 61% among 13–19 years age.

### Prevalence of *Schistosoma mansoni* infection and intensity of infections

**Fig 2:**
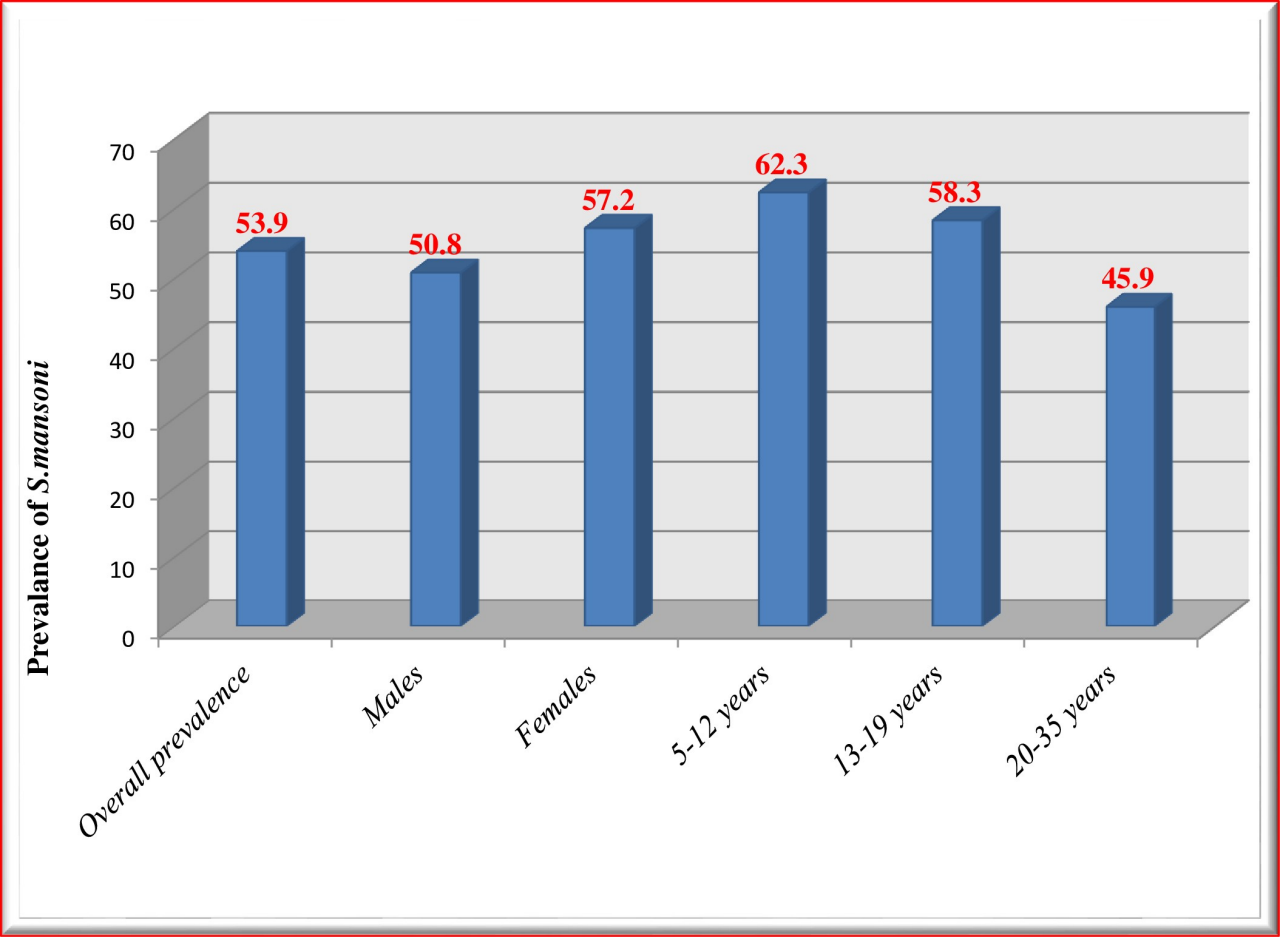
Prevalence of S. mansoni infections across sex and age groups.

**Fig 3:**
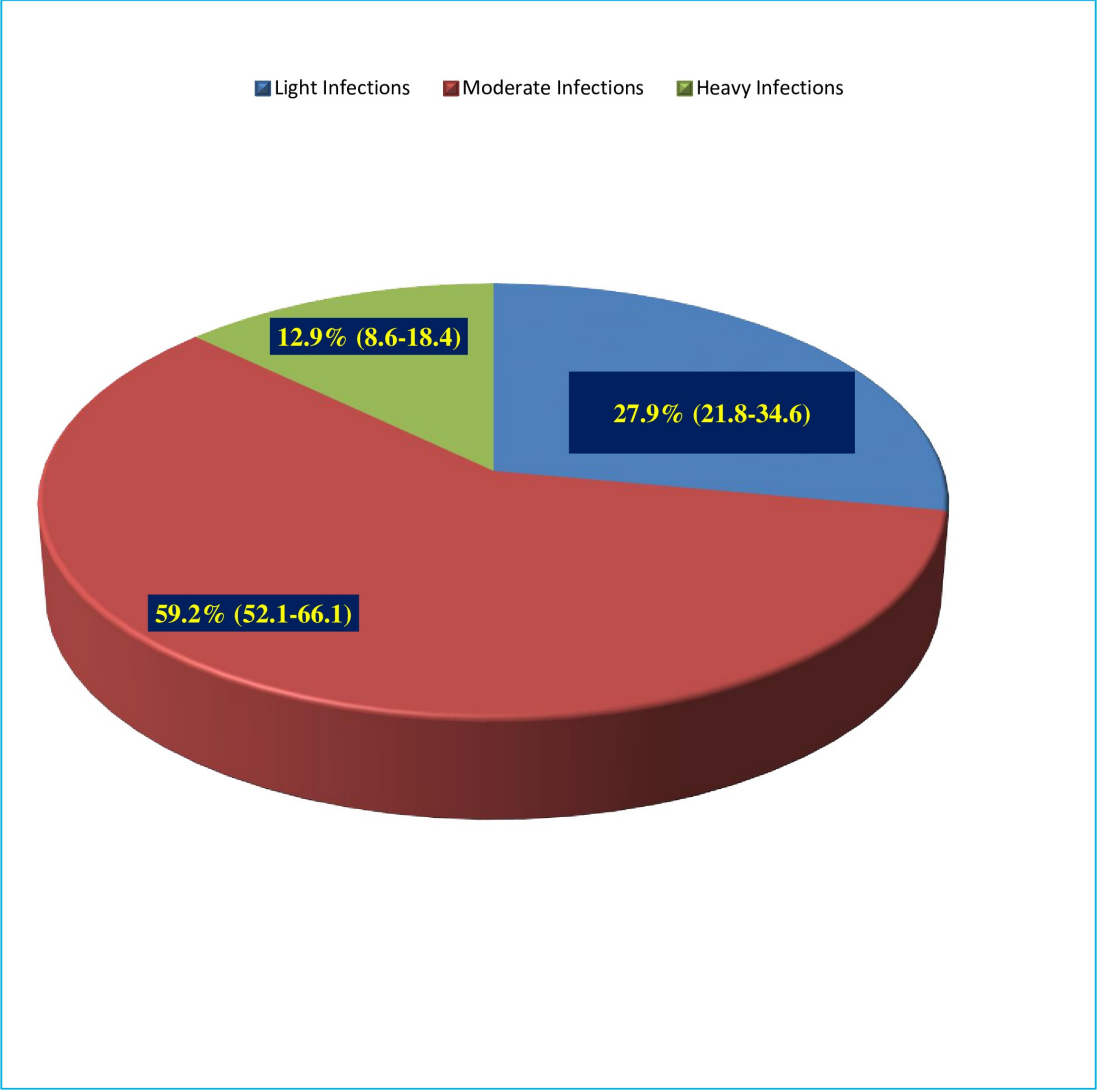
Intensity of S. mansoni infections.

The overall prevalence of *S*. *mansoni* infection was 53.9% (201/373) (Fig 2). Of which 59% (119/201) were moderate and 12.9% (12/201) were heavy infections (Fig 3). The prevalence was 50.8% among males and 57.2% among females. The prevalence was decreased with age (Fig 2). The overall arithmetic means egg count (ARMEC) was 204 EPG (min = 24epg and max = 1464epg).

### Factors associated with *S*. *mansoni* infection and intensities of infection

*Schistosoma mansoni* infection was significantly higher among the children below 12 years of age, both school-attending and non-attending. It was also high in daily laborers, but these were few (only 3). The intensity of infection was also higher in children (school-attending: 62.3% (86/138)) and non-attending: 58.3% (7/12). The intensity of infection was moderate to heavy in 73% (59/81) for those who had a history of deworming and very similar 72% (86/120) among those who had no history of deworming during the last MDA campaign (Table 2).

**Table 2:** *S*.*mansoni* infection status and intensity of infection and associated factors.

Variables	*S*. *mansoni* infection status				Total	P-value	*X*^2^
Occupation	Yes		No				
Farmer	31 (42.5%)		42 (57.5%)		73	0.024*	10.768
Student	138 (57.5%)		102 (42.5%)		240		
Salaried employee	17 (42.5%)		23 (57.5%)		40		
Non-attending school age	12 (75.0%)		4 (25.0%)		16		
Daily laborer	3 (75.0%)		1 (25.0%)		4		
Total	201 (53.9%)		172 (46.1%)		373		
Occupation	*S*.*mansoni* intensity of infection						
	Light	Moderate		Heavy			
Farmer	13 (41.9%)	16 (51.6%)		2 (6.4%)	31	0.418	7.674
Student	34 (24.6%)	86 (62.3%)		18 (13.0%)	138		
Salaried employee	6 (35.3%)	8 (47.1%)		3 (17.6%)	17		
Non-attending school age	3 (25.0%)	7 (58.3%)		2 (16.7%)	12		
Daily laborer	0 (0%)	2 (66.7%)		1 (33.3%)	3		
Total	56 (100%)	119 (100%)		26 (100%)	201		
History of deworming	*S*. *Mansoni* infection status						
	Yes		No				
Yes	81 (40.3%)		62 (36.0%)		143	0.455	0.709
No	120 (59.7%)		110 (60.0%)		230		
Total	201 (53.9%)		172 (46.1%)		373		
Intensity of infection	History of deworming						
	Yes		No				
Light	22 (27.2%)		34 (28.3%)		56	0.274	2.529
Moderate	52 (64.2%)		67 (55.8%)		119		
Heavy	7 (8.6%)		19 (15.8%)		26		
Total	81 (40.3%)		120 (59.7%)		201		
Age groups	*S*.*mansoni* infection status						
	Yes		No				
5–12	48 (62.3%)		29 (27.7%)		77	0.025*	7.360
13–19	81 (58.3%)		58 (41.7%)		139		
20–35	72 (45.9%)		85 (44.1%)		157		
Total	201 (53.9%)		172 (46.1%)		373		
Age groups	*S*.*mansoni* intensity of infection						
	Light	Moderate	Heavy				
5–12	12 (25.0%)	33 (68.8%)	3 (6.2%)		48	0.241	5.523
13–19	20 (24.7%)	50 (61.7%)	11 (13.6%)		81		
20–35	24 (33.3%)	36 (50.0%)	12 (16.7%)		72		
Total	56(27.9%)	119 (59.2%)	26 (12.9%)		201		

All values are number (%). x^2^. Chi-square. *Significant association (P < 0.05).

### Water body contact behavior and sources of water for bathing and domestic purposes

All of the study participants had a history of water contact activities including swimming, bathing, and washing in the rivers or streams. About 35% had habits of swimming, bathing, and /or washing all the days of the week, whereas the rest had reported three to five days of a week. Although all participants had habits of contact with rivers or streams, females and the majority of males in the two lower age groups had frequent contact.

The main sources of water for bathing were rivers and streams for all age groups. The main source of drinking water was a public hand pump. River and stream water sources were the main sources of drinking when they were out of their home. None of the participants had reported a habit of boiling water for drinking.

### Malacological result

A total of 623 snail samples were collected during the three seasons from Rivers and streams nearer to the three study villages (Table 3). Of them, 11.2%% (70/623) died during transportation. The rest of the snails (553) were exposed for shedding and 106 (19.2%) were shed cercariae. In January there was no *S*.*mansoni* snail intermediate host found from any of the water bodies. In March the first very small-sized snails were found in the rivers but were not collected. Whereas, in the same month, snails were found in the Sumala stream nearer to the Agallu Metti village from where 105 *B*. *pfeifferi* snails were collected and 11.4% (12) of them shedded cercariae. However, in May cercariae shedding *B*. *pfeifferi* snails were collected from all the water bodies. The snail infection rate was higher at Agallu Metti, from Sumala stream 59/189 (31.2%) and Metti river 32/194 (16.5%). Examination of the physical characteristics of the water bodies showed that water streams were full flooding and turbid in January, whereas they were clear and their volume was highly reduced in May. The water bodies were covered with large amounts of weeds, and other garbage: plastic, clothes, and fallen leaves on which the snails were found.

**Table 3:** Seasonal *Biomphalaria pfeifferi* snails’ distribution and infection status (n = 623).

Month of the survey	Districts (Villages)	Water bodies	Collected snails	Exposed snails for shedding	Died	Positives	Negatives	Remark
Mid-January	Agallu Metti	Metti River	0	0	0	0	0	No snails
	Chessega	Wau River	0	0	0	0	0	No snails
	Shimala	Jajeba River	0	0	0	0	0	No snails
	Agallu Metti	Sumala stream	0	0	0	0	0	No snails
Mid-March	Agallu Metti	Metti River	0	0	0	0	0	Very small sized few snails were seen
	Chessega	Wau River	0	0	0	0	0	
	Shimala	Jajeba River	0	0	0	0	0	
	Agallu Metti	Sumala stream	120	105	15 (12.5%)	12 (11.4%)	93 (89.6%)	light cercaria
Mid -May	Agallu Metti	Metti River	213	194	19 (8.9%)	32 (16.5%)	162 (83.5%)	Full of cercaria
	Chessega	Wau River	34	25	9 (26.5%)	2 (8%)	23 (92%)	Full cercaria
	Shimala	Jajeba River	46	40	5 (10.9%)	1 (2.5%)	39 (97.5%)	Full cercaria
	Agallu Metti	Sumala Stream	210	189	21 (10%)	59 (32%)	130 (68%)	Full of cercaria
Total			623	553	70 (11.2%)	106 (19.2%)	447 (80.8%)	

## Discussion

The overall prevalence of *S*.*mansoni* infection at present was 53.9% and most of the infections (59.2%) were moderate in intensity. The prevalence was about 60% among school-age children and about 50% among adult participants.

These rather isolated villages in the Abbey- and Didessa valleys of Western Ethiopia were treated in 1985–86 with a dramatic reduction in the prevalence and intensities of infection [13]. However, a survey in 1995, 10 years later, indicated that the effect might not be lasting [14]. In the present study done in 2019, the infection rates were back to the levels in the early 1980s, even though there has been an ongoing annual mass drug administration since 2015 in the area. This is another example of the lack of sustainability of isolated mass drug administrations without any other approach to control the other targets of the *Schistosoma* life cycle [9].

These findings indicate that the villages belong to a persistence hot spot area (PHS) under the category of high-risk communities. This might be due to frequent re-infection as everybody had water bodies contact behavior at least once a day [9]; MDA was only targeted at school children in school, a large fraction of the local *Schistosoma* infection burden remains untreated among non-attending school-age children and young adults [7]; inadequate sanitation [20], and in addition open-air defecation was common, whereas rivers and streams were commonly used sources of water in the area.

This study also indicates a seasonality of *schistosome* infection in these lowlands, similar to previous studies. The absence of the snails during the wet seasons corroborates with the study conducted in the same area by Gundersen *et al*. 30 years back [13]. The wash-out effect of high water velocity and flooding affect the density of *B*. *pfeifferi* in rivers as described by Gundersen *et al*. [13], which probably can explain why the reinfection after MDAs are slow and that the parasite loads are relatively low. So, since nature adds to the snail control and limits the re-infection rates, strategically placed MDA in hotspots of schistosomiasis transmission, as suggested by the SCORE group [9] could possibly keep the infection rates and parasite levels below the morbidity level.

The present results are similar to a previous study in Finchaa Sugar Estate in western Ethiopia, where Mekonnen et al reported a prevalence of 53.2% [21]. However, the present *S*.*mansoni* infection prevalence is higher than, the prevalence reported in north Gondar (33.7%) [22], in southern Ethiopia (13.35%) [23], and in Kenya (23.1%) [24], but lower than other prevalence reports: in northern Ethiopia (73.9%) [25], from different endemic localities of Ethiopia (69.7%) [26], and in the Democratic Republic of Congo (89.3%) [27]. These differences may be due to a difference in geographical and ecological variations, methods of diagnosis, the behavior, and awareness of the study participants, environmental sanitation, socioeconomic conditions, distribution and abundance of snail intermediate hosts, and variations in the study period among others [28].

The majority of the *S*.*mansoni* infections reported in this the study was moderate in intensity, which is consistent with findings from other parts of Ethiopia [25, 26], however, higher intensity of infection compared with many studies [21, 23, 25]. These differences may be due to a difference in geographical and ecological variations, methods of diagnosis, environmental sanitation, socioeconomic conditions, and variations in the study period among others [28].

Study participants in the age group of 5–12 years old were the most affected by *S*. *mansoni*, which is consistent with studies from; southwestern Ethiopia [29]. This might be due to frequent water-contact activities of this age group, as they pass most of their time playing in the rivers. Our observation also confirmed it.

The prevalence of *S*.*mansoni* infection was significantly different across occupations. School non-attending school-age children and daily laborers were commonly infected by *S*.*mansoni* than others. This finding is in line with a study in Uganda [30]. This may be due to the fact that these groups were not covered by the MDA program and frequent contact with cercariae infected water bodies.

There were similar levels of infection and intensity of infection among those who had a history of deworming, compared to those who had not received previous deworming during the fifth round MDA. This might be due to frequent re-infection as everybody had water bodies contact behavior, at least once a day and furthermore, because cure rates with PZQ are estimated to be 70–90%, even with excellent coverage, MDA will leave a proportion of adult worms and all juvenile worms alive [9].

This high prevalence of *S*.*mansoni* infection and high infection rates among the intermediate snail hosts in most of the rivers and streams in the study area, which tributes the Abbey river and the new lake that formed by the Grand Ethiopian Renascence Dam (GERD) in the area may worsen the public health impact of this infection. The spatial and temporal distribution of water level and sediment in the middle and lower reaches of the rivers will be changed, and as a consequence, there will be a series of changes in the ecological environment of the marshland along the rivers and the adjacent areas of the lake. This will have impacts on the schistosomiasis prevalence in the middle and lower reaches of the rivers. The lake that formed will increase the activities of people in the area and correspondingly increases the risk of contact with infected water, thus increasing the risk of schistosomiasis infection in the area [12]. Thus, urgent integrated schistosomiasis control strategies are needed.

Although the current study was carefully conducted, we are aware of its limitations. First, the parasitological screening was performed using only the Kato-Katz method which could lead to underestimation of the reports. Secondly, possibly high-risk individuals of selected households may have been in the field during the visits, which could also lead to underestimation of the reports. Thirdly, recall bias and confusion of schistosomiasis MDA with other deworming campaigns may under or overestimate MDA coverage reports.

## Conclusions

*Schistosoma mansoni* infection is still a public health problem in the study areas, despite control efforts already 30 years ago and present MDA in the last years. School-age children need special attention, because of the highest prevalence of parasitic among these. The infection status of snail intermediate hosts was also high, especially at the end of the dry season. So, special attention involving an integrated approach is needed in the area for the success of global schistosomiasis control and elimination efforts. We suggest making the MDA campaign just early after the rainy season, when the snails are washed away. In addition, since school-based MDA alone, not seems effective: expansion from school-based to community-wide treatment programs and the combined use of multiple interventions like improving water, sanitation, and hygiene (WASH) through the provision of clean water plus sanitation and reduction of water contact and possible snail control efforts’ to prevent reinfection is needed.

## Supporting information

S1 Data All the data used in the manuscript. (XLSX)
